# *In vitro* modeling of experimental succinic semialdehyde dehydrogenase deficiency (SSADHD) using brain-derived neural stem cells

**DOI:** 10.1371/journal.pone.0186919

**Published:** 2017-10-20

**Authors:** Kara R. Vogel, Garrett R. Ainslie, Erwin E. Jansen, Gajja S. Salomons, Jean-Baptiste Roullet, K. Michael Gibson

**Affiliations:** 1 Experimental and Systems Pharmacology and Pharmacotherapy, College of Pharmacy, Washington State University, Spokane, WA; 2 Metabolic Unit, Department of Clinical Chemistry, VU University Medical Center, Neuroscience Campus, Amsterdam, The Netherlands; University of Nebraska Medical Center, UNITED STATES

## Abstract

We explored the utility of neural stem cells (NSCs) as an *in vitro* model for evaluating preclinical therapeutics in succinic semialdehyde dehydrogenase-deficient (SSADHD) mice. NSCs were obtained from *aldh5a1*^*+/+*^ and *aldh5a1*^*-/-*^ mice (aldh5a1 = aldehyde dehydrogenase 5a1 = SSADH). Multiple parameters were evaluated including: (1) production of GHB (γ-hydroxybutyrate), the biochemical hallmark of SSADHD; (2) rescue from cell death with the dual mTOR (mechanistic target of rapamycin) inhibitor, XL-765, an agent previously shown to rescue *aldh5a1*^*-/-*^ mice from premature lethality; (3) mitochondrial number, total reactive oxygen species, and mitochondrial superoxide production, all previously documented as abnormal in *aldh5a1*^*-/-*^ mice; (4) total ATP levels and ATP consumption; and (5) selected gene expression profiles associated with epilepsy, a prominent feature in both experimental and human SSADHD. Patterns of dysfunction were observed in all of these parameters and mirrored earlier findings in *aldh5a1*^*-/-*^ mice. Patterns of dysregulated gene expression between hypothalamus and NSCs centered on ion channels, GABAergic receptors, and inflammation, suggesting novel pathomechanisms as well as a developmental ontogeny for gene expression potentially associated with the murine epileptic phenotype. The NSC model of SSADHD will be valuable in providing a first-tier screen for centrally-acting therapeutics and prioritizing therapeutic concepts of preclinical animal studies applicable to SSADHD.

## Introduction

**Fig 1:**
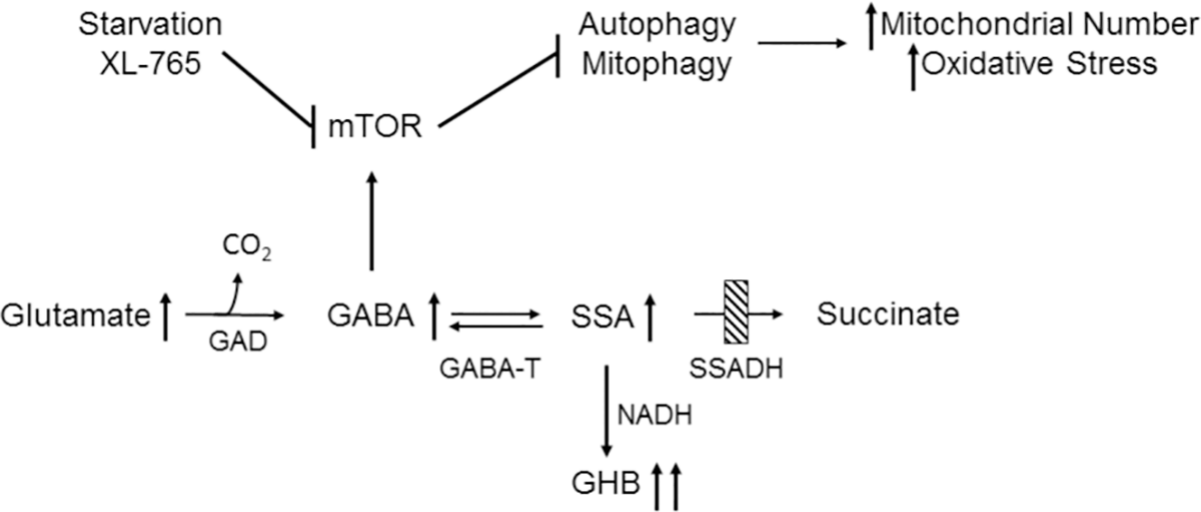
Interrelationships of GABA metabolism, and the effect of GABA on mTOR (mechanistic target of rapamycin). Upward arrows indicate elevations of metabolites seen in both human and murine SSADHD. Bars indicate blockade. Abbreviations: GAD, glutamate decarboxylase; GABA-T, GABA-transaminase; SSA, succinic semialdehyde; GHB, γ-hydroxybutyrate; SSADH, succinic semialdehyde dehydrogenase; NADH, reduced nicotinamide dinucleotide.

Succinic semialdehyde dehydrogenase (SSADH; aldehyde dehydrogenase 5a1, ALDH5A1) deficiency (SSADHD), the most prevalent disorder of GABA metabolism, is an orphan, autosomal-recessive disorder. Metabolically, two neuropharmacologically active species, GABA (4-aminobutyric acid) and the GABA-derivative, γ-hydroxybutyric acid (GHB), accumulate. The former is the major central inhibitory neurotransmitter, inasmuch as 1/3^rd^ of central synapses utilize it for inhibitory neurotransmission. GHB, present in the central nervous system at approximately 1% of parent GABA, associates with a pharmacological profile that features alteration of dopamine release and reuptake [1]. GHB is used as an agent to induce absence seizures, as a euphoric agent and drug to perpetrate sexual assault, and a clinically-approved therapeutic for narcolepsy [2, 3] (Fig 1). Beyond its approved FDA indication for narcolepsy, GHB is a Schedule I controlled substance.

The neurological phenotype of SSADHD includes developmental delay, neuropsychiatric morbidity, the absence of developed speech, hypotonia and variable seizures [3]. Targeted therapeutics are lacking, and the majority of therapeutic agents are symptomatically administered. Based upon studies in the corresponding knockout mouse model (*aldh5a1*^*-/-*^ mice), two clinical trials have been initiated. In one, the use of the sulfonated amino acid, taurine, was without clinical benefit despite its demonstrated efficacy in the mouse model with respect to lifespan extension [4–6]. A second trial is underway employing the GABA(B) receptor antagonist SGS-742, a drug which effectively mitigated aberrant spike-wave discharge in the murine model [7].

The mouse model of SSADH deficiency has provided valuable avenues from which to explore additional preclinical therapeutics targeting SSADHD, including NCS-382 (a GHB receptor antagonist), neuroactive steroids, and agents that inhibit the mechanistic target of rapamycin [4, 8–11]. The SSADHD mouse has facilitated the collection of outcome data such as survival, electrophysiology, metabolite levels and gene expression [3, 10, 12]. The latter has focused our interest on the capacity of mTOR inhibitors to extend the survival of *aldh5a1*^*-/-*^ mice in vivo. Additionally, we have identified perturbations in the expression of various genes related to GABA and glutamate signaling in this model. Examination of these changes has provided novel insight into disease pathophysiology and highlighted new routes of investigation.

While the *aldha5a1*^*-/-*^ mouse is a valuable tool in the identification of pharmacological targets for SSADHD, an ideal screening paradigm would include an in vitro system to reduce the number of animals used, in addition to time and cost constraints. One such approach would be to develop an *aldh5a1*^*-/-*^ brain-derived cell line that could be used to screen therapeutics for their ability to correct key cellular abnormalites observed in SSADHD such as GHB accumulation, oxidative stress, aberrant mitochondrial function and dysregulated gene expression. Brain-derived neural stem cell (NSCs) appeared to be a logical if not an attractive choice for the development of such a screening tool. NSCs are primary progenitors to neurons and glial cells in the embryonic, neonatal and adult brain [13], and as such could be used to study SSADHD brain pathophysiology in vitro when compared to wild-type (WT) NSCs. Further, NSCs can be maintained in a proliferative stage almost indefinitely while retaining the capacity to differentiate into neurons, astrocytes or oligodendrocytes with validated cell culture protocols [14].

In brief, NSCs have a unique in vitro modeling potential of the SSADHD brain, a model suitable for the study of early developmental changes of the central nervous system and for the rapid screening of compounds with potential therapeutic activity. Accordingly, we derived and cultured NSCs from *aldh5a1*^*-/-*^ mice to assess their modeling potential of the SSADHD brain. Then, we determined if these cells presented the major biochemical and mitochondrial features of SSADHD by comparing these features with those of wild-type (*aldh5a1*^*+/+*^) NSCs and intact *aldh5a1*^*-/-*^ mouse brain. Last, we had reported that mTOR inhibitors significantly increased the lifespan of *aldh5a1*^*-/-*^ mice while improving brain cell biology. We thus sought to reproduce these in vivo findings exposed *aldh5a1*^*-/-*^ NSCs with the same drugs that showed phenotypic rescue in vivo. Our findings indicate that *aldh5a1*^*-/-*^ NSCs express most of the markers of the SSADHD mouse brain and hold significant value in the screening of potential therapeutics applicable to human SSADHD.

## Results

### Confirmation of elevated GHB levels in cell culture media and donor selection

**Fig 2:**
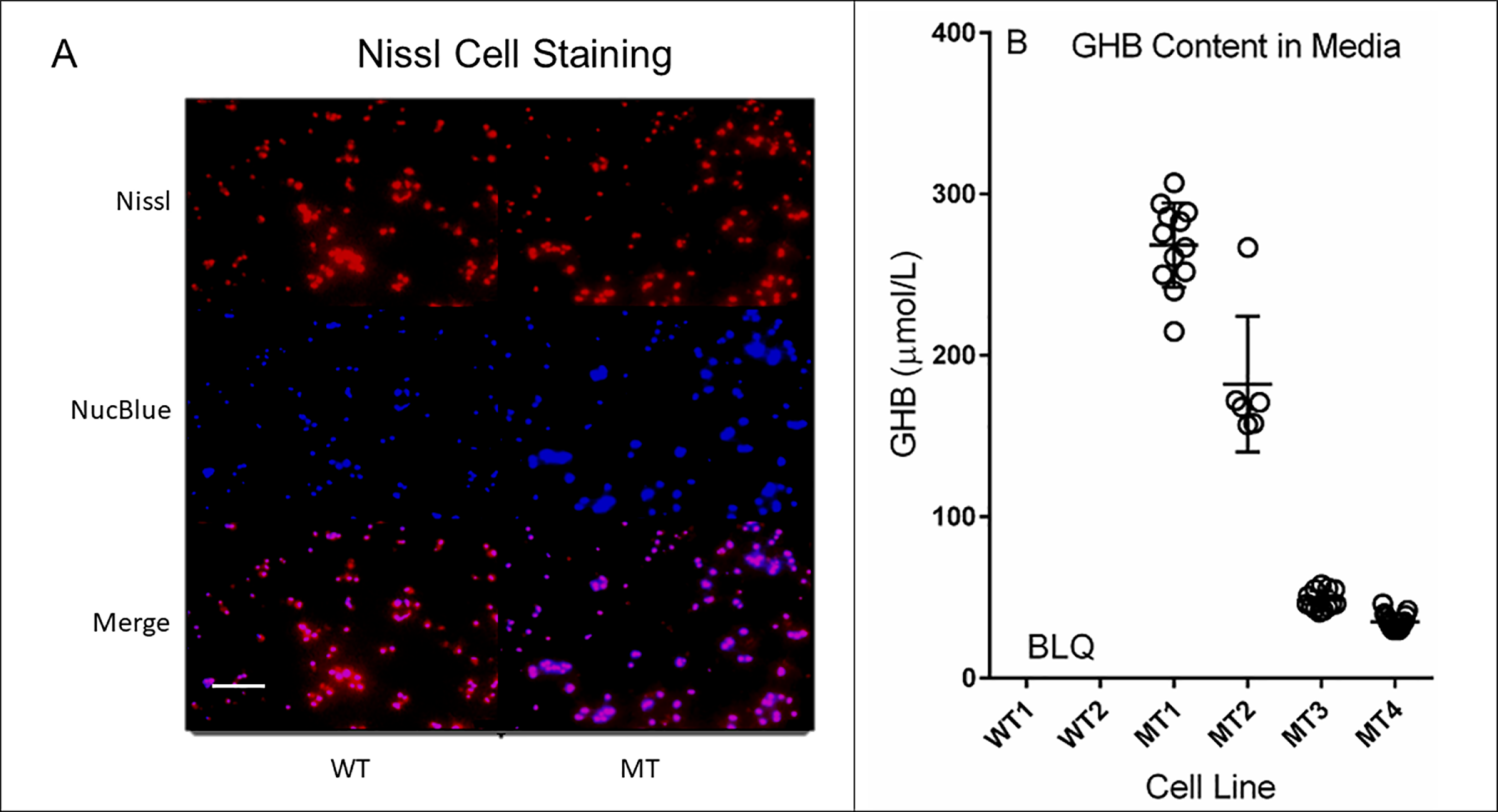
Nissl staining of neural stem cells (NSCs) and cell culture medium concentration of GHB as a function of cell line genotype. To confirm that NSCs retained features of neural cells, Nissl stain (red) was initially undertaken, and co-localization with a nuclear stain (blue) was performed to confirm that cells were of neuronal origin (A), although the Nissl stain does not differentiate mature neurons from progenitors. Scale bar = 50 μm (white bar). GHB concentration in cell culture medium is also displayed as a function of donor cell line (B). Media were collected for 24 h following addition of fresh medium and quantified for GHB content employing isotope-dilution gas chromatography-mass spectrometry [29]. GHB content was normalized to the recovery of ^2^H_6_-GHB, the latter added to each medium sample as internal standard stable isotope. The lower limit of quantification for this assay was 30 μM; all wild-type samples were below the limit of quantification (BLQ). Error bars display mean and SD. Abbreviations: WT, wild-type; MT, mutant. Statistical analysis employed one-way ANOVA (F (3, 42) = 378.9; p < 0.0001).

To establish the metabolic phenotype, neural stem cells (NSCs) were isolated from the cortex and hippocampus of P1 wildtype (N = 4) and mutant (N = 4) mice, cultured and expanded as described in *Methods*. GHB levels were measured in the media of all *aldh5a1*^*-/-*^ donors and in two wildtype donors, the latter selected based on visual inspection of proliferation rate, morphology and general health in culture. GHB media levels were below the limit of quantification (<30 μM) in all wildtype cultures (n = 6–7 wells/culture) (Fig 2B). GHB levels measured in the media of mutant NSCs varied, with donors 1 and 3 having >6-fold increase relative to wildtype cultures (Fig 2B). Conversely, donors 7 and 9 produced far lower levels of GHB (one-way ANOVA, F (3, 42) = 378.9; p<0.0001). For subsequent experiments, wildtype donor 13 (WT) and mutant donor 1 (MT) were used as experimental platforms. To most closely mimic SSADHD in vivo, we chose to examine NSC lines with the highest accumulation of GHB among all clonal NSCs. The derivation of these cells as NSCs was subsequently confirmed using Nissl stain in addition to markers of stemness (Fig 2A),including Nestin and SOX2 (S1 Fig; S1 Table) which was observed in both donors 3–4 passages following the experiments described.

### Cell viability as a function of mTOR inhibition

**Fig 3:**
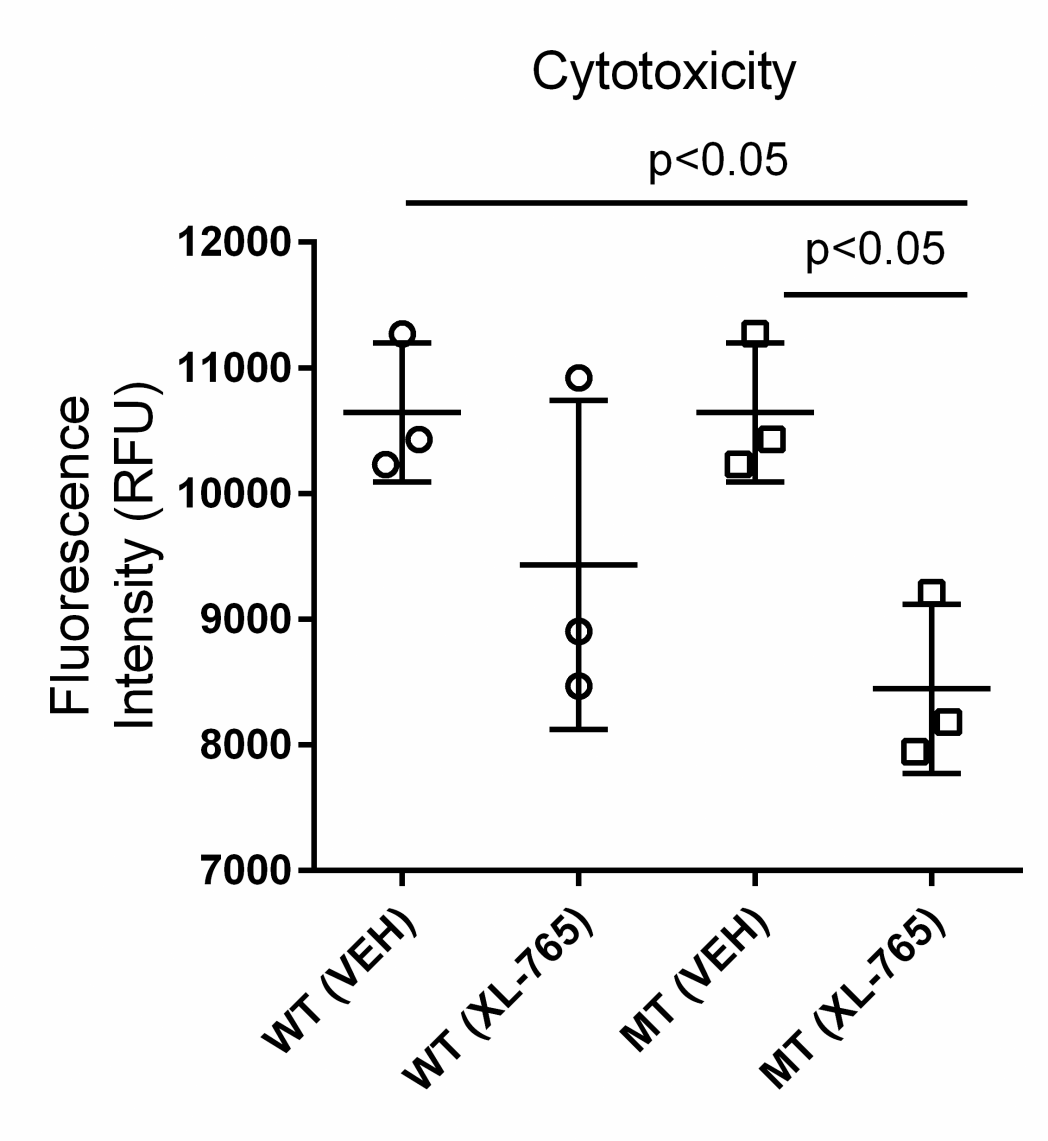
Fluorescence detection of cellular toxicity as a function of starvation or treatment with the mTOR inhibitor, XL-765. NSCs were exposed to vehicle (0.01% DMSO) or XL-765 (see Methods) for 24 h, then starvation conditions were implemented for 6 h followed by fluorescence quantification to assess cytotoxicity. Each symbol (square) represents the measurement of each biological replicate. Each horizontal line reflects the mean of the 3 independent experiments and error bars represent SD. When present, XL-765 was used at 10 nM in DMSO (0.01%) for 24 h, otherwise vehicle matched controls were employed. Note that a lower value within genotype represents decreased cytotoxicity. Abbreviations: RFU, relative fluorescence units; WT, wild-type; MT, mutant; VEH (0.01% DMSO); XL-765, treatment with XL-765. Statistical analysis employed one-way ANOVA with post-hoc analysis (F (3, 8) = 4.895; p < 0.05).

We have previously demonstrated that the dual PI3K/mTORC1/2 inhibitor, XL-765, significantly extended the lifespan of *aldh5a1*^*-/-*^mice, while simultaneously resulting in significant improvement in body weight in these animals [10]. Accordingly, we evaluated the effect of this dual mTOR inhibitor on the degree of cellular viability for NSCs derived from wild-type and mutant mice grown under basal (starvation) conditions (Fig 3). XL-765 did not improve the cellular viability of wt cells, yet resulted in a significant reduction of cytotoxicity for mutant cells, providing evidence of a protective effect as previously observed with *aldh5a1*^*-/-*^ mice (one-way ANOVA (F (3,8) = 4.895; p<0.05).

### Evaluation of mitochondrial number, parameters of oxidative stress, and ATP levels under starvation conditions and mTOR inhibition in NSCs

Based on earlier results of mitochondrial dysfunction in *aldh5a1*^*-/-*^ mice, including increased parameters of oxidative stress and elevated organelle abundance [9, 11, 15, 16], we examined similar parameters in NSCs. Readouts for mitochondrial oxidative stress were examined after 6, 12, 24, and 36 h of cell growth in metabolic starvation conditions (complete medium without B27, EGF and FGF). Starvation conditions were chosen for these studies since we would expect to see normal cells undergo autophagy, a process that is perturbed in *aldh5a1*^*-/-*^ mice [9, 11, 15, 16].

**Fig 4:**
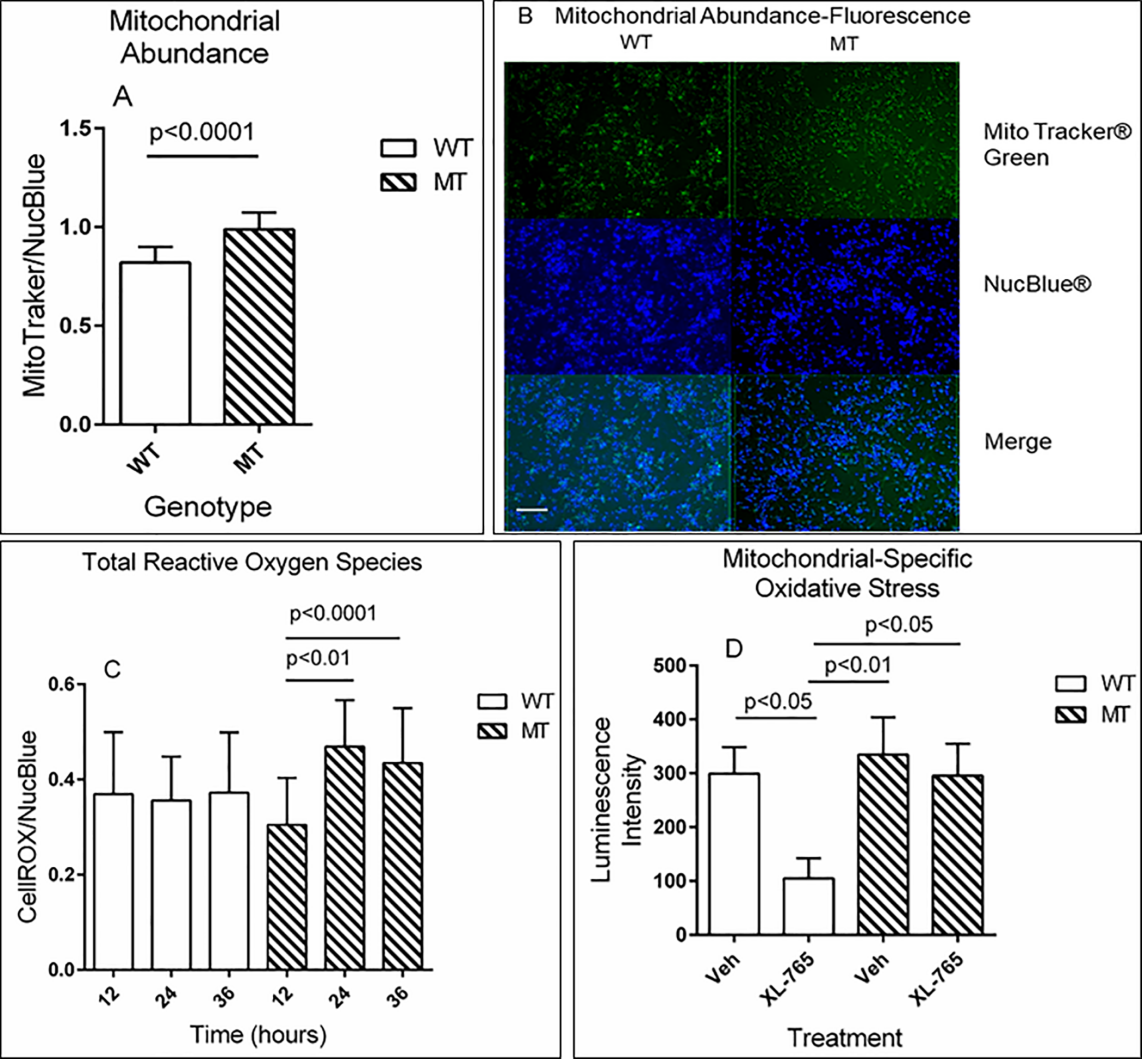
Mitochondrial abundance and oxidative stress parameters in NSCs. Mitotracker^TM^ fluorescent staining was employed to assess mitochondrial abundance.Quantitation of fluorescent microscopy images in which MitoTracker signal (green) was normalized to nuclear stain (blue, NucBlue) (A) and representative fluorescence images employed for data accrual (B; scale bar = 50 μm (white)). CellROX^TM^ staining was used to assess overall degree of oxidative stress (total reactive oxygen species), and different durations of treatment with starvation media (12–36 hours) (C). Mitochondrial specific oxidative stress was determined with a luminescence assay (MitoSox^TM^ system), in the presence and absence of 10 nM XL-765 (normalized to cell viability) (D). All values represent the mean of 3 replicates with error bars denoting SD. Data for Fig 4A assessed using a two-tailed t test. For Fig 4C, a one-way ANOVA with post-hoc analysis was used within genotype (wild-type, (F (2, 51) = 12.5; p = ns: mutant, F (2, 51) = 12.5; p<0.0001) and for Fig 4D across genotypes (F (3, 8) = 10.72, p<0.01). Abbreviations employed were as described in Fig 3.

Mitochondrial staining was significantly stronger in mutant cells than in wt cells after 6 h in starvation conditions, indicating the presence of a greater number of mitochondria in SSADHD NSCs than in controls (Fig 4A; two-tailed t test, p<0.0001). Representative fluorescent images corresponding to these analyses are shown in Fig 4B.Oxidative stress levels, measured by CellROX^TM^ fluorescent staining demonstrated significant differences between mutant and wt NSCs (Fig 4C). These levels increased with time and were maximum at 24 hrs in mutant NSCs, whereas they remained unchanged in wild-type cultures (Fig 4C; one-way ANOVA within genotype: wild-type F (2, 51) = 12.5, p = ns and mutant F (2, 51) = 12.5, p<0.0001)). As a further exploration of oxidative stress, we then examined mitochondrial superoxide (O_2_^-^) in NSCs, and the potential protective activity of mTOR inhibition with XL-765. Superoxide production was similar in both wt and mutant NSCs in the absence of XL-765 (Fig 4D; vehicle). It was significantly reduced by XL-765 (~3 fold) in wt NSCs but remained unchanged in mutant NSCs (Fig 4D; one-way ANOVA across genotypes F (3, 8) = 10.72, p<0.01).

**Fig 5:**
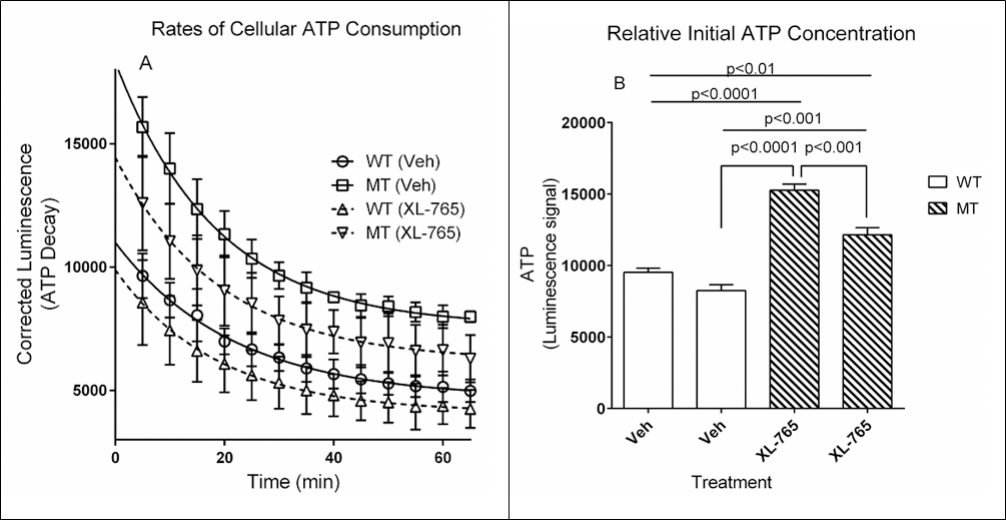
Relative ATP consumption rates and initial ATP concentration in NSCs. ATP concentrations were detected with an ATP-sensitive luminescent probe and monitored for 65 minutes to evaluate the rate of ATP consumption (A; indicating relative concentrations at that time point). Initial relative ATP concentration (B) between genotype and treatment. Data represents the mean of 3 replicates and error bars represent SD. XL-765 was employed at 10 nM in vehicle (Veh; 0.01% DMSO) for 24 h. Data in Fig 4A were analyzed using two-way ANOVA for half-life, as well as effects of time and treatment/genotype. This revealed interaction (half-lives;t_1/2_ in min.) for ATP consumption that were not significantly different between mutant (starvation conditions, 58.6 [50.6–69.5] and XL-765, 56.0 [44.4–75.7]) and wild-type (starvation conditions, 56.0 [48.9–65.2] and XL-765, 58.6 [48.1–75.0]) following 24 hr of growth/treatment (F (36, 104) = 0.8699; p = ns). Conversely, effects of time and treatment/genotype were significant (time, F (12, 104) = 41.85, p<0.0001; treatment/genotype, F (3, 104) = 177.4; p<0.0001). Data for Fig 5B was evaluated with a one-way ANOVA and post hoc analysis (F (3, 12) = 56.19; p<0.0001. Abbreviations employed were as in Figs 3 and 4.

To extend these studies, we next quantified ATP levels in NSCs, as well as time-dependent decay of ATP concentration, both as an effect of starvation conditions and in the presence of the dual mTOR inhibitor XL-765 (Fig 5A and 5B). The data show that ATP levels are higher in mutant cells than in wt cells at all time points during the 60 minute recording time. The higher ATP level in the mutant cell line is also consistent with enhanced mitochondrial number, as previously predicted [16]. Measurement of individual relative ATP concentrations in Fig 5A revealed a typical first-order decay profile. The presence of the dual mTOR/PI3K inhibitor, XL-765, resulted in a significant lowering of ATP level for both, consistent with mTOR inhibition leading to down-regulation of both autophagy and mitophagy and clearing of accumulated mitochondria. Data in Fig 5A were evaluated with a two-way ANOVA for ATP t_1/2_, as well as the effect of treatment and genotype. ATP t_1/2_ values were not significantly different between mutant (starvation conditions, 58.6 [50.6–69.5] and XL-765, 56.0 [44.4–75.7]) and wild-type (starvation conditions, 56.0 [48.9–65.2] and XL-765, 58.6 [48.1–75.0]) following 24 hr of growth/treatment (F (36, 104) = 0.8699; p = ns). Conversely, effects of time and treatment/genotype were significant in Fig 5A (time, F (12, 104) = 41.85, p<0.0001: and treatment/genotype F (3,104) = 177.4, p<0.0001. The data for Fig 5B was evaluated employing a one-way ANOVA (F (3, 12) = 56.19, p<0.0001.

### Evaluation of epilepsy gene expression in NSCs, and P1 and P20 hypothalami, derived from *aldh5a1*^*+/+*^ and *aldh5a1*^*-/-*^ mice and NSCs

In these studies, we sought to characterize the molecular characteristics of mutant NSCs. We focused on genes involved in oxidative stress and neurotransmission, the expression of which was reported as abnormal in *aldh5a1*^*-/-*^ mice [10, 11]. We evaluated NSCs, as well as hypothalami derived from both P1 (postnatal day of life 1) and P20 (postnatal day of life 20) of *aldh5a1*^*-/-*^ and *aldh5a1*^*+/+*^ mice. Our rationale for this approach was based upon the observation that gene expression should differ over the course of development [17], and that the majority of NSCs derive from the hypothalamus [18]. This approach also afforded a novel opportunity to contrast gene expression in P1 NSCs with the developmental ontogeny of gene expression in tissue extracts prepared from matching brain regions of the murine model.

**Fig 6:**
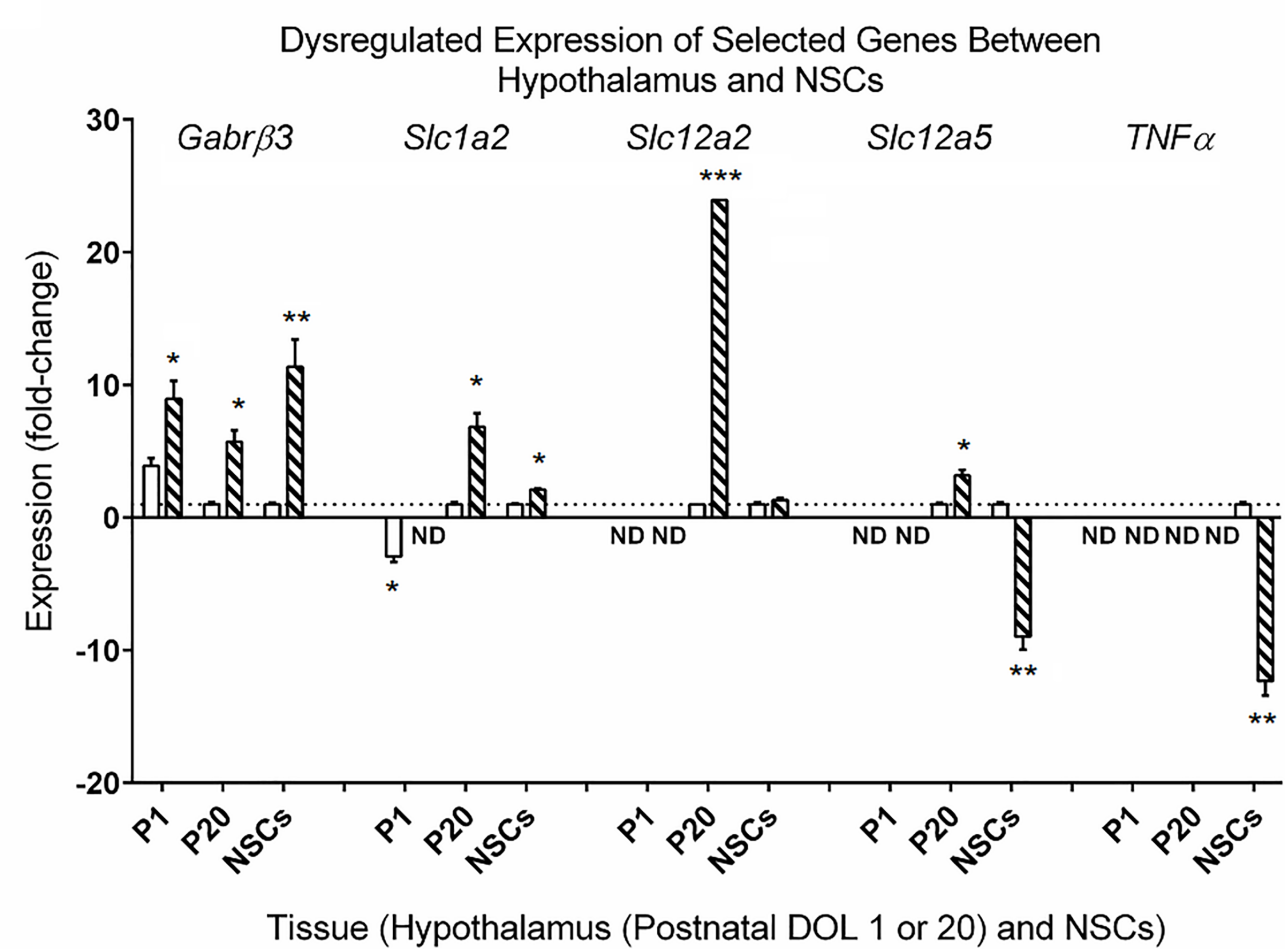
Selected genes dysregulated in NSCs as compared to homogenates of isolated hypothalami. Neural stems cells were harvested following 24 h of culture in 6-well plates; hippocampi were dissected from P1 and P20 (postnatal day of life) *aldh5a1*^*+/+*^ (white boxes) and *aldh5a1*^*-/-*^ (black boxes) mice. Shown are *Gabrb3*, the β3 subunit of the GABA_B_ receptor, solute carriers (*Slc*) *1a2*, *12a2* and *12a5*, respectively, representing family members of the solute carrier organic acid transporter family, and tumor necrosis factor α (TNFα). Gene expression was measured via quantitative reverse transcription-polymerase chain reaction (RT-PCR). NSC expression levels were normalized to wild-type NSCs, and hippocampal extract results were normalized to P20 wild-type mice. Bars denote the mean ± SD of 3–4 biological replicates. ND, denotes that expression was not detectable. Note the developmental differences in the solute carrier transporters as a function of both tissue age and NSCs, underscoring a high degree of ontogeny. Conversely, up-regulation of the β3 subunit of the GABA_B_ receptor was consistent regardless of tissue or age. Statistical analysis, Student’s two-tailed *t* test; *p<0.05; **p<0.01; ***p<0.001.

Using a standard array for characterization of genes under/over-expressed in epileptic syndromes (see Methods), we identified six genes dysregulated at least 4-fold in *aldh5a1*^*-/-*^ NSC cultures relative to wild-type. The differential expression of five of these genes is depicted (Fig 6). In NSCs and *aldh5a1*^*-/-*^ hypothalamus (P1, P20), *Gabrb3* (the β3 subunit of the GABA_A_ receptor) was highly and consistently overexpressed relative to *aldh5a1*^*+/+*^ mice. Solute carriers, including sodium-dependent glutamate/aspartate transporter 2 (*Slc1a2*), sodium-potassium-chloride cotransporter 1 (NKCC1; *Slc12a2*), and neural potassium chloride cotransporter (*Slc12a5*), were all expressed at very low, or undetectable, levels in P1 mouse hypothalamus (Fig 6.) [19] (“SLC12A5 Solute Carrier Family 12 Member 5 [Homo Sapiens (Human)]—Gene—NCBI” 2017; “SLC12A2 Solute Carrier Family 12 Member 2 [Homo Sapiens (Human)]—Gene—NCBI” 2017). Expression of these transporters increased significantly by DOL20, all >3-fold higher in *aldh5a1*^*-/-*^ vs. *aldh5a1*^*+/+*^ mice. For NSCs, the trend of solute carrier expression was similar for *Slc1a2* and *Slc12a2*, but displayed an inverse relationship with *Slc12a5*, most likely representing the balance of channel functions involved in depolarization vis-à-vis hyperpolarization. Finally, we found a significant down-regulation of TNFα in mutant NSCs in comparison to that of wild-type (Fig 6), providing the first evidence for aberrant inflammation in SSADH deficiency. This is of interest since TNFα represents a key cytokine in systemic inflammation and the acute phase reaction.

## Discussion

In the current study, our objective was to develop and undertake preliminary validation of an in vitro model system in which to study preclinical therapeutics applicable to SSADHD. Generally speaking, pharmacotoxicity evaluations employ peripheral in vitro models, including hepatic and/or renal cells. On the other hand, therapeutics for neurological disorders must, by definition, be brain penetrant and resident. Accordingly, we have undertaken the development of NSCs from *aldh5a1*^*-/-*^ mice as a potential in vitro modeling system. The utility of such a model is many-fold: (1) less expensive than modeling in *aldh5a1*^*-/-*^ mice, whose lifespan is truncated; (2) the capacity for high-throughput analysis; (3) bypass of peripheral metabolic processes with focus on neuropharmacology; (4) potential to “prioritize” therapeutics applicable to SSADHD to move forward to preclinical animal studies; and (5) in vitro modeling of the developing brain, with ability to study the impact of candidate drugs on neurogenesis. Here, we have assessed several parameters previously characterized in *aldh5a1*^*-/-*^ mice (including GHB excretion, oxidative stress, mitochondrial function, and gene expression) in NSCs using the dual mTOR inhibitor XL-765 as our investigative agent, the latter known to be an effective therapeutic in *aldh5a1*^*-/-*^ mice [10].

Initially, we confirmed the neuroprogenitor status of wt and mutant NSCs with positive immunostaining for Nestin and SOX2 (S1 Fig). We then demonstrated that mutant cells synthesize and accumulate GHB as predicted from our in vivo studies (Fig 2). GHB accumulation was variable depending on the mutant cell line. Such metabolic variability in cell culture has been noted [20], perhaps related to minor alterations in DNA content, epigenetic considerations, senescence endpoints or simply confluency stage. Despite this, the difference between all *aldh5a1*-deficient NSC cell lines tested and those derived from wild-type cell lines was readily demonstrated since no GHB was produced and detected in wild-type cells and their conditioned media. Moving forward in a testing system, we opted to examine the deficient NSC line with the highest GHB excretion (Fig 2). We were unable to quantify GABA in cell culture medium of NSCs as the signal was too low, not unexpected with an amino acid species whose ionic nature likely precluded cellular efflux.

We have recently provided extensive evidence for the activation of the mechanistic target of rapamycin (mTOR) by elevated GABA in SSADHD [9, 11, 15, 16]. In these studies, we showed that mTOR activation by GABA blocks autophagy, increases mitochondrial numbers and impairs mitochondrial function (see Fig 1). This effect can be overridden by drugs and conditions (rapamycin, Torin 1, Torin 2, starvation), effects that have been highlighted in the *aldh5a1*^*-/-*^ mouse model [10, 11]. Based upon this rationale, we opted to characterize mitochondrial number and function, and the effect of mTOR inhibition, the latter shown to significantly mitigate early lethality in *aldh5a1*^*-/-*^ mice [10].

We first examined cellular cytotoxicity under starvation conditions in the presence and absence of XL-765, and confirmed the protective effects of XL-765 (Fig 3). This outcome in *aldh5a1*^*-/-*^ NSCs is consistent with the positive in vivo effects of XL-765 in *aldh5a1*^*-/-*^ mice [10]. Mitochondrial number and function were next examined. Imaging of cells cultured in metabolic starvation conditions revealed a significant increase in fluorescent MitoTracker staining for mitochondria, normalized to (NucBlue) cell number (Fig 4A), again consistent with increased mitochondrial numbers observed in liver and brain derived from *aldh5a1*^*-/-*^ mice [11, 16]. These results were extended to reveal a significantly increased level of reactive oxygen species in mutant NSCs in response to starvation, increase that was not found in wild-type NSCs (Fig 4B), again consistent with enhanced mitochondrial numbers in mutant cells. We then examined the potential of XL-765 to override the dysfunction of oxidative stress parameters observed in Fig 4B and determined the effect of XL-765 on superoxide production. As shown in Fig 4D, XL-765 significantly decreased superoxide production in wild-type NSCs. A trend toward attenuation of superoxide production was also observed in mutant NSCs, consistent with results seen in brain derived from *aldh5a1*^*-/-*^ mice treated with the mTOR inhibitor rapamycin [16]. However, the effect of XL-765 in mutant cells was not statistically significant. The apparent resistance to the protective effect of XL-765 in mutant NSCs may be explained by cells having a higher basal mTOR signaling activity to overcome than wt cells. Such possibility could be tested with higher concentrations of the drug and longer exposure time in future experiments. As a final examination of mitochondrial number and function, we quantified initial ATP levels in NSCs, as well as rate of decay with time (Fig 5). As predicted, ATP levels were significantly higher in mutant NSCs as compared to wild-type, consistent with enhanced mitochondrial numbers. These levels were significantly attenuated with mTOR inhibition employing XL-765, in both cell lines, which is also consistent with the capacity of XL-765 to reduce/clear mitochondrial numbers in NSCs.

Recent work has revealed that the use of mTOR inhibitors in *aldh5a1*^*-/-*^ mice can lead to significant improvement in gene expression abnormalities associated with neurotransmission, as well as oxidative stress [10, 11]. Accordingly, as a final readout of the utility of NSCs, we implemented a limited gene expression analysis. Since NSCs were isolated from P1 hypothalami we decided to compare NSC gene expression with gene expression in P1 hypothalami. We also compared NSC gene expression with gene expression in P20 hypothalami, a developmental stage closer to the postnatal age at which mutant mice die. Hypothalamus was chosen as a region of particular interest as electrophysiological studies indicate that GABA remains excitatory in this brain region post-development [13]. We found that the β3 subunit of the GABA_A_ receptor (Fig 6) was significantly overexpressed throughout development, and correspondingly increased in NSCs. Although these results were consistent, they contrasted with early results in *aldh5a1*^*-/-*^ mice (untreated with mTOR inhibitors) which revealed a low level but significant down-regulation of this same GABA_A_ receptor subunit β [10]. However, the latter were performed with whole brain extracts, and not isolated hypothalamus, emphasizing the importance of regional mapping of gene expression in our model system. Nonetheless, the consistent and significant perturbation of this subunit of the GABA_A_ receptor provides evidence that, in addition to enhanced mitochondria production and increased levels of intracellular oxidative stress, NSCs also display the predicted anomalies indicative of disordered GABA metabolism with respect to GABAergic receptor function. Experimental evidence has shown this subunit is regulated by repressor element 1 (RE1)-silencing transcription factor (REST)-mediated gene silencing as a result of status epilepticus [21]. We further observed differential regulation of solute carriers *1a2* (EAAT2), *12a2* (NKCC1) and *12a5* (KCC2) as a function of developmental age, and for *12a5* there was an inverse relation with *12a2* in hypothalamus.

In the developing brain, NKCC1 is upregulated, while KCC2 levels are low. These two cation chloride cotransporters are reciprocally regulated by osmosensitive WNK kinases, the resulting expression of NKCC1 and KCC2 impacting polarity of GABA_A_Rs and glycine receptors [22]. GABA_A_ receptor activation can lead to both de- and hyperpolarization of the neuron, with hyperpolarization being the predominant “behavior” in mature neurons, and depolarization being observed early in development (all dependent upon the intracellular Cl^-^ concentration, which is higher early in development) [23]. Considering the perplexing association of high GABA concentration with high seizure frequency in SSADHD [11], it remains possible that SSADHD neurons stay “immature” and have a constitutively high intracellular chloride concentration. If this were the case, GABA would thus cause depolarization in SSADHD neurons and seizures. Whether this actually occurs remains to be experimentally investigated.

Importantly, we found a significant down-regulation of TNFα in mutant NSCs, providing a link between GABA, mTOR and the stress-response associated with inflammatory cytokines (Fig 6). mTOR is known to be regulated by TNFα through a variety of stress-dependent transcriptional factors [24–26]. This represents the first evidence of alterations in parameters of inflammation in SSADH deficiency. However, mTORC1 positively regulates TNFα [27, 28], such that we would predict increases in TNFα in mutant cells, and not down-regulation. This paradox may again reflect the progenitor nature of NSCs, as well as the potential neural immaturity that we suggest may occur in SSADHD. Studies are underway in P1 and P20 hypothalami to determine if TNFα expression, as well as other cytokines, are upregulated in *aldh5a1*^*-/-*^ tissues, as expected. If this were the case, this situation would parallel our findings between tissues and NSCs for *Slc12a5*.

In conclusion, we have demonstrated the validity of in vitro modeling of the SSADHD brain with NSCs and established the usefulness of these cells in the screening of promising therapeutics. Although drugs such as Torin 1 and 2, NCS-382 and SGS-742 have already demonstrated preclinical utility in *aldh5a1*^*-/-*^ mice [4, 7, 10], other therapeutic strategies are actively under investigation in our laboratory, including therapeutics with neurosteroid intermediates as well as novel allosteric GABA_A_ receptor activators. The availability of NSCs as a model system for drug screening will enable a more rapid evaluation of these preclinical therapeutics prior to moving forward to animal studies.

## Materials and methods

### Isolation and culture of mouse-derived neural stems cells (NSC)

At postnatal day one of life (P1), pups (*aldh5a1*^*+/+*^ *and aldh5a1*^*-/-*^) were sacrificed, and the brain gently eviscerated into Hibernate media supplemented with penicillin/streptomycin and B27 medium (Thermo Fisher, Waltham, MA, USA). The cortex and hippocampal region from *aldh5a1*^*+/+*^ and *aldh5a1*^*-/-*^ brains, following genotyping (see below), were then dissociated with Versene (Thermo Fisher) in warm media (37°C). Cells were maintained in complete medium consisting of Neurobasal^®^ A Medium (Gibco Cat. No. 21103; Thermo Fisher) supplemented with B-27^®^ Serum-Free Supplement, GlutaMAX^TM^ (Gibco Cat. No. 35050–061), antibiotic-antimycotic solution (Gibco Cat. No. 15240), FGF-basic (AA 10–155; recombinant human (bFGF), Cat. No. PHG0024), and EGF (recombinant human epidermal growth factor (Millipore, Darmstadt, Germany). Cells were initially expanded in 35-mm diameter tissue culture dishes, then into 100-mm dishes coated with poly-L-ornithine (Sigma, Cat. No. P3655). Confluent cells were passed using Versene as described above. Tail biopsies were obtained from each donor and genotype was determined as previously described [10]. The dual mTOR/PI3K inhibitor, XL-765, was purchased from Tocris Biosciences (Bristol, UK), and used as the sole therapeutic agent in our studies. For all cell studies, passage 1–3 were consistently employed. All studies involving vertebrate animals were approved by the WSU IACUC (ASAF 4232, 4276).

### Nissl stain and immunochemistry

NeuroTrace^TM^ fluorescent Nissl Stain was purchased from Molecular probes (Eugene, OR; cat: N-21483), and provided as a DMSO stock (1 ml). The working stain solution was prepared as a 0.1% Tritron-X100 solution in phosphate buffered saline (PBS; pH 7.4, 10 ml). For the final stain, 0.05 ml of Nissl stock was diluted to 10 ml of Triton/PBS buffer. Culture medium was removed from cells to be stained, followed by addition of 100 μl of cell staining solution, incubation and fluorescent imaging using a high red filter. Nissl substance (rough endoplasmic reticulum) appears red due to the staining of ribosomal RNA, and represents an overall measure of neural structure. NSCs were also stained with a panel of specific antibodies to confirm their undifferentiated neuroprogenitor status. The cells were cultured in 24-well poly-L-ornithine coated black plates with μClear^TM^ bottom, stained with NucBlue live and then fixed with 4% formaldehyde in DPBS. Immunostaining was performed after permeabilization with 0.1% Triton X-100 in DPBS, incubation in a blocking solution (3% BSA in DPBS) for 60 minutes, and several rinses with DPBS. Neuroprogenitor cells are expected to express Nestin and Sox2 proteins(Ivanov and Hei, 2015). All primary antibodies were prepared based on the manufacturer’s recommended dilution and incubation times for IHC (S1 Table). SOX2antibodies were conjugated with DyLight 488, eFluor570 and eFluor570, respectively. Nestin was unconjugated and was visualized using a goat anti-mouse IgG Secondary DyLight 488 antiserum (3 h incubation). Cells were imaged using an InCell High Content Analyzer 2200 (GE Healthcare Life Sciences, Little Chalfont, United Kingdom) at 20x magnification.

### Quantification of GHB in neural stem cells

Cell media were collected (total volume, 200–500 μl; n ≥ 6 well/donor) and diluted at 1:1 v/v in 6% sulfosalicylic acid. Metabolite quantitation in NSC media was performed using isotope dilution methodology and gas chromatography-mass spectrometry (GC-MS) with ^2^H_6_-GHB as internal standard [29]. The limit of quantification (LOQ) was ~30 μM GHB in NSC media.

### Fluorescence cocktail employed to assess changes in organelle number, biomarkers of oxidative stress, and perturbations of autophagy

For these experiments, cells were cultured in 24-well plates (seeding density of 0.25 x10^6^ cells/cm^2^). Following 24 hours in culture, cells were supplemented with media favoring starvation through omission of growth factors (bFGF and EGF) and B27. This media devoid of bFGF, EGF, and B27 is referred to as the ‘basal’ or ‘starvation’ media throughout the text and is employed to induce autophagy, a process which is dysregulated in *aldh5a1*^*-/-*^ mice and responsive to correction by mTOR inhibitors [10, 11], such as XL-765. The fluorescence cocktail employed to assess biomarkers listed above included: NucBlue^®^ (2 drops/ml), Mitotracker^®^ Green (1 nM or 1 μl/10 ml), LysoTracker^®^ Red (75 nM or 0.75 μl/10 ml) and CellROX^®^ Deep Red (5 μM or 20 μL/10 ml media; final volume achieved with Basal media). This cocktail was applied to individual cultures of cells at 5, 11, and 23 h following the initiation of starvation conditions, with fluorescence imaging performed at 6, 12, and 24 hours on a Leica fluorescent microscope (40x magnification). NucBlue was employed to normalize values to total cell number (based upon nuclei staining). Mitotracker green, Lysotracker Red and CellROX Deep Red measures were blank corrected and subsequently normalized to NucBlue (Table 1).

**Table 1:** Reagents employed for in vitro characterization of neural stem cells.

Stain or Kit	Measured Parameter	Supplier	Product number
NeuroTrace^TM^	Nissl substance	Molecular Probes	N21483
NucBlue^®^ Live Ready Probes^®^ reagent	Nucleus	Molecular Probes	R37605
MitoTracker^®^ Green FM	Mitochondria	Molecular Probes	M7514
CellRox^®^ Deep Red	Reactive oxygen species	Molecular Probes	C10422
Mitochondrial ToxGlo^TM^	Cytotoxicity and Cellular ATP	Promega	G8000
ApoLive Glo^TM^	Cell viability	Promega	G6410
MitoSOX^TM^	Mitochondrial Superoxide	Molecular Probes	M36008

### Cytotoxicity and ATP consumption assay in neural stem cells

NSCs cells were plated onto white 96-well plates with clear bottoms (VWR; cat:89136–852) in complete medium, and subjected to treatment for 24 h. At least 3 wells containing cells were left untreated to serve as blanks for background signal correction. The Mitochondrial ToxGlo^TM^ kit (Promega, Madison, WI; Table 1) was used according to the manufacturer’s instructions to sequentially measure cytoxicity (fluorescence measurements) and cellular ATP levels (luminescence measurements). Cumulative outcomes using the ToxGlo assay included the initial ATP levels (reflected from the initial luminescence measurements), as well as the rate of ATP consumption (initial rate, and half-life (t_1/2_); Table 1). All measurements were performed using a BioTek Synergy microplate reader.

### Mitochondrial superoxide levels in neural stem cells

Mitochondrial superoxide levels were measured in NSCs using the MitoSOX^TM^ Red dye (Molecular Probes, M36008; Thermo Fisher Scientific) (Table 1). A 5 mM stock solution was prepared in DMSO and diluted 100-fold in complete media to a final concentration of 5 μM (0.1% DMSO) and fluorescence was measured via microplate reader with blank values subtracted.

### Collection of hypothalamus from P1 and P20 *aldh5a1*^*+/+*^ and *aldh5a1*^*-/-*^ mice for gene expression analysis

SSADH-deficient mice (*aldh5a1*^*-/-*^ mice) were sacrificed at P1 and P20 and brains rapidly removed onto a cold plate for dissection. Tissues were snap frozen in liquid nitrogen and kept on dry ice until storage at minus 80⁰C. DNA was extracted from tail biopsies and amplified by three primer PCR and gel electrophoresis to establish genotype [10, 11, 30]. Trizol (Invitrogen) was used to extract RNA per manufacturer protocol, and the Turbo DNase Kit (Ambion) was used to remove DNA contaminants.

### Measurement of gene expression in neural stem cells and hypothalami using qRT-PCR (reverse transcription-polymerase chain reaction)

Preparation of cell and tissue extracts, and gene expression analysis, followed previously published methods [11, 30]. In brief, Sybr green (Biorad) reactions were prepared as a master mix with 50 ng of cDNA/10 μl/well aliquoted into prearrayed and validated (per MIQE guidelines) BioRad pathway finder plates (Epilepsy M384; PrimePCR^TM^ SABioscience) [31]. A BioRad CFX384 real-time PCR with BioRad CFX manager v3.0 software was employed for data acquisition, normalization, and statistical analysis.

### Statistical analysis

Evaluators were not blinded to genotype of cell lines, nor to treatment conditions. Prior to data analysis, the Gaussian (or non-Gaussian) skew of all data was visually assessed, and an absence of outliers confirmed for replicates. Data is presented in bar graphs and represents mean ± 1 SD. Statistical analyses employed two-tailed t test or ANOVA (one- and two-way, as appropriate) followed by Tukey post-hoc testing relative to vehicle or genotype controls. Statistical analyses were conducted using GraphPad Prism 6 (San Diego, CA, USA), and both p value and F statistic reported in Results and in corresponding Figure Legends. For gene expression studies, data were normalized to GAPDH (glyceraldehyde-3-phosphate dehydrogenase) and Tbp (TATA binding protein), employed as housekeeper genes. These normalized values of mutant NSCs were compared to wildtype NSCs, and normalized values from P1 and P20 mutant, and P1 wild-type mouse brains, were normalized to P20 wildtype brains. For all gene expression analysis a predefined cutoff of 4-fold was used to assess differences from control samples.

## Supporting information

S1 Fig Representative immunostaining of NSCs (wild-type and mutant) for SOX and Nestin, confirming the neural progenitor status of our cell lines. (TIF)

S1 Table Antibodies and incubation conditions used for immunohistochemistry. (DOCX)

## References

[1] Maitre M, Klein C, Mensah-Nyagan AG. Mechanisms for the specific properties of γ-hydroxybutyrate in brain. Med Res Rev. 2016;36: 363–88. doi: 10.1002/med.21382 26739481 10.1002/med.21382

[2] Attri SV, Singhi P, Wiwattanadittakul N, Goswami JN, Sankhyan N, Salomons GS, et al Incidence and geographic distribution of succinic semialdehyde dehydrogenase (SSADH) deficiency. JIMD Rep. 2016 11 5. [Epub ahead of print] 10.1007/8904_2016_14 PMC5509553 27815844

[3] Malaspina P, Roullet JB, Pearl PL, Ainslie GR, Vogel KR, Gibson KM. Succinic semialdehyde dehydrogenase deficiency (SSADHD): Pathophysiological complexity and multifactorial trait associations in a rare monogenic disorder of GABA metabolism. Neurochem Int. 2016;99: 72–84. doi: 10.1016/j.neuint.2016.06.009 27311541 10.1016/j.neuint.2016.06.009 PMC5028283

[4] Gupta M, Greven R, Jansen EE, Jakobs C, Hogema BM, Froestl W, et al Therapeutic intervention in mice deficient for succinate semialdehyde dehydrogenase (gamma-hydroxybutyric aciduria). J Pharmacol Exp Ther. 2002;302(1): 180–7. 12065715 10.1124/jpet.302.1.180

[5] Pearl PL, Schreiber J, Theodore WH, McCarter R, Barrios ES, Yu J, et al Taurine trial in succinic semialdehyde dehydrogenase deficiency and elevated CNS GABA. Neurology 2014;82: 940–4. doi: 10.1212/WNL.0000000000000210 24523482 10.1212/WNL.0000000000000210 PMC3963004

[6] Schreiber JM, Pearl PL, Dustin I, Wiggs E, Barrios E, Wassermann EM, et al Biomarkers in a taurine trial for succinic semialdehyde dehydrogenase deficiency. JIMD Rep. 2016;30: 81–87. doi: 10.1007/8904_2015_524 27338723 10.1007/8904_2015_524 PMC5110443

[7] Pearl PL, Gibson KM, Cortez MA, Wu Y, Carter Snead O 3rd, Knerr I, et al Succinic semialdehyde dehydrogenase deficiency: lessons from mice and men. J Inherit Metab Dis. 2009;32: 343–52. doi: 10.1007/s10545-009-1034-y 19172412 10.1007/s10545-009-1034-y PMC2693236

[8] Ainslie GR, Gibson KM, Vogel KR. A pharmacokinetic evaluation and metabolite identification of the GHB receptor antagonist NCS-382 in mouse informs novel therapeutic strategies for the treatment of GHB intoxication. Pharmacol Res Perspect. 2016;4: e00265 doi: 10.1002/prp2.265 27891231 10.1002/prp2.265 PMC5115179

[9] Vogel KR, Ainslie GR, Jansen EE, Salomons GS, Gibson KM. Torin 1 partially corrects vigabatrin-induced mitochondrial increase in mouse. Ann Clin Transl Neurol. 2015;2: 699–706. doi: 10.1002/acn3.200 26125044 10.1002/acn3.200 PMC4479529

[10] Vogel KR, Ainslie GR, Gibson KM. mTOR inhibitors rescue premature lethality and attenuate dysregulation of GABAergic/glutamatergic transcription in murine succinate semialdehyde dehydrogenase deficiency (SSADHD), a disorder of GABA metabolism. J Inherit Metab Dis. 2016a; 39:877–886. 27518770 10.1007/s10545-016-9959-4 PMC5114712

[11] Vogel KR, Ainslie GR, Jansen EE, Salomons GS, Gibson KM. Therapeutic relevance of mTOR inhibition in murine succinate semialdehyde dehydrogenase deficiency (SSADHD), a disorder of GABA metabolism. Biochim Biophys Acta. 2016b; 1863: 33–42. 27760377 10.1016/j.bbadis.2016.10.009 PMC5154833

[12] Jansen EE, Vogel KR, Salomons GS, Pearl PL, Roullet JB, Gibson KM. Correlation of blood biomarkers with age informs pathomechanisms in succinic semialdehyde dehydrogenase deficiency (SSADHD), a disorder of GABA metabolism. J Inherit Metab Dis. 2016;39: 795–800. doi: 10.1007/s10545-016-9980-7 27686230 10.1007/s10545-016-9980-7 PMC5115636

[13] Migaud M, Batailler M, Segura S, Duittoz A, Franceschini I, Pillon D. Emerging new sites for adult neurogenesis in the mammalian brain: a comparative study between the hypothalamus and the classical neurogenic zones. Eur J Neurosci. 2010;32: 2042–52. doi: 10.1111/j.1460-9568.2010.07521.x 21143659 10.1111/j.1460-9568.2010.07521.x

[14] Ivanov DP, Al-Rubai AJ, Grabowska AM, Pratten MK. Separating chemotherapy-related developmental neurotoxicity from cytotoxicity in monolayer and neurosphere cultures of human fetal brain cells. Toxicol In Vitro. 2016;37: 88–96. doi: 10.1016/j.tiv.2016.09.007 27622579 10.1016/j.tiv.2016.09.007

[15] Vogel KR, Ainslie GR, Schmidt MA, Wisor JP, Gibson KM. mTOR Inhibition Mitigates Molecular and Biochemical Alterations of Vigabatrin-Induced Visual Field Toxicity in Mice. Pediatr Neurol. 2017;66: 44–52. doi: 10.1016/j.pediatrneurol.2016.09.016 27816307 10.1016/j.pediatrneurol.2016.09.016 PMC5866057

[16] Lakhani R^,^ Vogel KR, Till A, Liu J, Burnett SF, Gibson KM, et al Defects in GABA metabolism affect selective autophagy pathways and are alleviated by mTOR inhibition. EMBO Mol Med. 2014;6: 551–566. doi: 10.1002/emmm.201303356 24578415 10.1002/emmm.201303356 PMC3992080

[17] Schneider E, Dittrich M, Böck J, Nanda I, Müller T, Seidmann L, et al CpG sites with continuously increasing or decreasing methylation from early to late human fetal brain development. Gene. 2016;592: 110–8. doi: 10.1016/j.gene.2016.07.058 27468947 10.1016/j.gene.2016.07.058

[18] Goodman T, Hajihosseini MK. Hypothalamic tanycytes-masters and servants of metabolic, neuroendocrine, and neurogenic functions. Front Neurosci. 2015;9:387 doi: 10.3389/fnins.2015.00387 26578855 10.3389/fnins.2015.00387 PMC4624852

[19] Watanabe M, Fukuda A. Development and regulation of chloride homeostasis in the central nervous system. Front Cell Neurosci. 2015 9 24;9:371 doi: 10.3389/fncel.2015.00371 26441542 10.3389/fncel.2015.00371 PMC4585146

[20] Vernardis SI, Goudar CT, Klapa MI. Metabolic profiling reveals that time related physiological changes in mammalian cell perfusion cultures are bioreactor scale independent. Metab Eng. 2013;19: 1–9. doi: 10.1016/j.ymben.2013.04.005 23680586 10.1016/j.ymben.2013.04.005

[21] Goldberg EM, Coulter DA. Mechanisms of epileptogenesis: a convergence on neural circuit dysfunction. Nat Rev Neurosci. 2013;14: 337–49. doi: 10.1038/nrn3482 23595016 10.1038/nrn3482 PMC3982383

[22] Chamma I, Chevy Q, Poncer JC, Lévi S. Role of the neuronal K-Cl co-transporter KCC2 in inhibitory and excitatory neurotransmission. Front Cell Neurosci. 2012;6: 5 doi: 10.3389/fncel.2012.00005 22363264 10.3389/fncel.2012.00005 PMC3282916

[23] Kilb W. Development of the GABAergic system from birth to adolescence. Neuroscientist 2012;18; 613–630. doi: 10.1177/1073858411422114 21952258 10.1177/1073858411422114

[24] Pietrocola F, Izzo V, Niso-Santano M, Vacchelli E, Galluzzi L, Maiuri MC, et al Regulation of autophagy by stress-responsive transcription factors. Semin Cancer Biol. 2013;23: 310–22. doi: 10.1016/j.semcancer.2013.05.008 23726895 10.1016/j.semcancer.2013.05.008

[25] Ding K, Wang H, Xu J, Lu X, Zhang L, Zhu L. Melatonin reduced microglial activation and alleviated neuroinflammation induced neuron degeneration in experimental traumatic brain injury: Possible involvement of mTOR pathway. Neurochem Int. 2014;76: 23–31. doi: 10.1016/j.neuint.2014.06.015 24995391 10.1016/j.neuint.2014.06.015

[26] Wang K. Autophagy and apoptosis in liver injury. Cell Cycle. 2015;14: 1631–42. doi: 10.1080/15384101.2015.1038685 25927598 10.1080/15384101.2015.1038685 PMC4614283

[27] Vangan N, Cao Y, Jia X, Bao W, Wang Y, He Q, et al mTORC1 mediates peptidoglycan induced inflammatory cytokines expression and NF-κB activation in macrophages. Microb Pathog. 2016;99: 111–118. doi: 10.1016/j.micpath.2016.08.011 27524262 10.1016/j.micpath.2016.08.011

[28] He Z, He X, Chen Z, Ke J, He X, Yuan R, et al Activation of the mTORC1 and STAT3 pathways promotes the malignant transformation of colitis in mice. Oncol Rep. 2014;32: 1873–80. doi: 10.3892/or.2014.3421 25174408 10.3892/or.2014.3421

[29] Gibson KM, Aramaki S, Sweetman L, Nyhan WL, DeVivo DC, Hodson AK, et al Stable isotope dilution analysis of 4-hydroxybutyric acid: an accurate method for quantification in physiological fluids and the prenatal diagnosis of 4-hydroxybutyric aciduria. Biomed Environ Mass Spectrom. 1990;19: 89–93. 2407302 10.1002/bms.1200190207

[30] Ivanov VN, Hei TK. Regulation of viability, differentiation and death of human melanoma cells carrying neural stem cell biomarkers: a possibility for neural trans-differentiation. Apoptosis. 2015;20: 996–1015. doi: 10.1007/s10495-015-1131-3 25953317 10.1007/s10495-015-1131-3 PMC4449821

[31] Bustin SA, Benes V, Garson JA, Hellemans J, Huggett J, Kubista M, et al The MIQE guidelines: minimum information for publication of quantitative real-time PCR experiments. Clin Chem. 2010;55: 611–22. 10.1373/clinchem.2008.112797 19246619

