# Supplementary figures and images for: In vitro modeling of experimental succinic semialdehyde dehydrogenase deficiency (SSADHD) using brain-derived neural stem cells
Source: PLoS One. 2017 Oct 20;12(10):e0186919. doi: 10.1371/journal.pone.0186919 (PMC5650182; doi:10.1371/journal.pone.0186919)

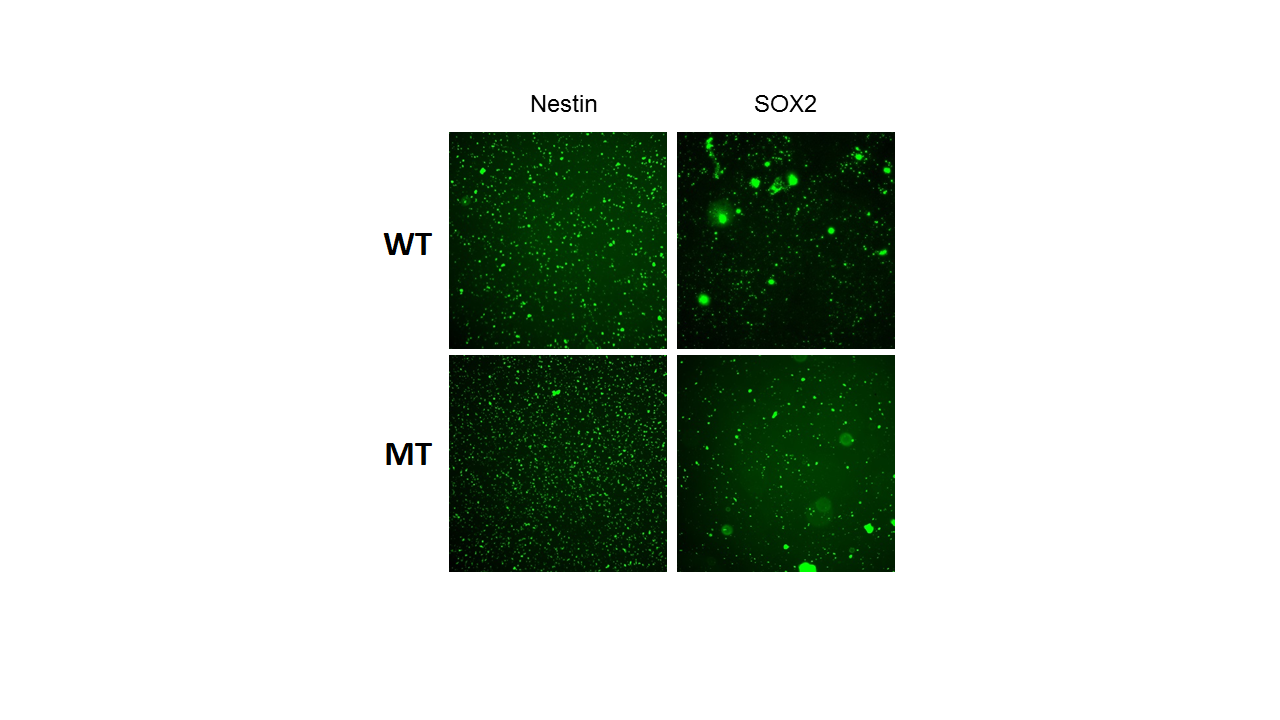

Supplement: S1 Fig — (TIF) [file pone.0186919.s001.TIF]
