# Supplementary material for: In vitro modeling of experimental succinic semialdehyde dehydrogenase deficiency (SSADHD) using brain-derived neural stem cells
Source: PLoS One. 2017 Oct 20;12(10):e0186919. doi: 10.1371/journal.pone.0186919 (PMC5650182; doi:10.1371/journal.pone.0186919)
Supplement: S1 Table — (DOCX) [file pone.0186919.s002.docx]

**S1 Table** (Vogel et al)

PONE-D-17-15493R2_FTC

| Protein | Antibody | Conjugate | Dilution | Incubation time (h) |
| --- | --- | --- | --- | --- |
| Nestin | Nestin monoclonal, mouse reactive | none | 1:100 | 3 (primary)  1 (secondary) |
| SOX2 | Monoclonal, mouse reactive | DyLight 488 | 1:100 | 1 |
